# Using quantitative systems pharmacology to evaluate the drug efficacy of COX-2 and 5-LOX inhibitors in therapeutic situations

**DOI:** 10.1038/s41540-018-0062-3

**Published:** 2018-08-03

**Authors:** Christoph Thiel, Ines Smit, Vanessa Baier, Henrik Cordes, Brigida Fabry, Lars Mathias Blank, Lars Kuepfer

**Affiliations:** 1 Institute of Applied Microbiology (iAMB), Aachen Biology and Biotechnology (ABBt), RWTH Aachen University, Worringerweg 1, 52074 Aachen, Germany; 2 European Molecular Biology Laboratory, European Bioinformatics Institute (EMBL-EBI), Wellcome Genome Campus, Hinxton, Cambridge CB10 1SD UK

## Abstract

A quantitative analysis of dose–response relationships is essential in preclinical and clinical drug development in order to optimize drug efficacy and safety, respectively. However, there is a lack of quantitative understanding about the dynamics of pharmacological drug–target interactions in biological systems. In this study, a quantitative systems pharmacology (QSP) approach is applied to quantify the drug efficacy of cyclooxygenase-2 (COX-2) and 5-lipoxygenase (5-LOX) inhibitors by coupling physiologically based pharmacokinetic models, at the whole-body level, with affected biological networks, at the cellular scale. Both COX-2 and 5-LOX are key enzymes in the production of inflammatory mediators and are known targets in the design of anti-inflammatory drugs. Drug efficacy is here evaluated for single and appropriate co-treatment of diclofenac, celecoxib, zileuton, and licofelone by quantitatively studying the reduction of prostaglandins and leukotrienes. The impact of rifampicin pre-treatment on prostaglandin formation is also investigated by considering pharmacokinetic drug interactions with diclofenac and celecoxib, finally suggesting optimized dose levels to compensate for the reduced drug action. Furthermore, a strong correlation was found between pain relief observed in patients as well as celecoxib- and diclofenac-induced decrease in prostaglandins after 6 h. The findings presented reveal insights about drug-induced modulation of cellular networks in a whole-body context, thereby describing complex pharmacokinetic/pharmacodynamic behavior of COX-2 and 5-LOX inhibitors in therapeutic situations. The results demonstrate the clinical benefit of using QSP to predict drug efficacy and, hence, encourage its use in future drug discovery and development programs.

## Systems pharmacology: PBPK/PD modeling at the whole-body scale

Drug efficacy is governed by both pharmacokinetics at the whole-body level and pharmacodynamic responses at the cellular scale. A team around Lars Kuepfer at Germany’s RWTH Aachen University applied a quantitative systems pharmacology (QSP) approach to assess the therapeutic potential for a set of drugs in the treatment of pain and inflammatory diseases. To this end, the authors integrated a cellular network model of arachidonic acid metabolism into physiologically based pharmacokinetic models to obtain a quantitative understanding about the dynamics of pharmacological drug–target interactions. The integrated multiscale model was used to address prototypical questions of drug development programs such as dose finding, drug–drug interactions and assessment of therapeutic efficacy. The results of the study demonstrate the benefits of using QSP in future drug development programs.

## Introduction

Novel drugs need to be efficacious and safe and the validation of both properties is mandatory for market authorization. If new drug candidates fail to show either therapeutic efficacy or safety the corresponding pharmaceutical development programs are consequently discontinued. A truly mechanistic assessment of physiological and biochemical processes governing the drug exposure and the resulting drug action is hence beneficial to improve the still high attrition rates of new drug candidates.^1^ However, such an in-depth understanding is hard to achieve given the complexity of physiological processes simultaneously interacting at different levels of biological organization.

Quantitative systems pharmacology (QSP) bears the promise to significantly support pharmaceutical development programs through a mechanistic consideration of processes underlying drug absorption, distribution, metabolism, and excretion (ADME) as well as the resulting drug action itself.^2–4^ As such, QSP aims for a detailed description of drug pharmacokinetics (PK) and, simultaneously, drug pharmacodynamics (PD). QSP approaches^5–8^ may therefore be used to quantitatively assess complex PK/PD behavior of drugs toward a systems-level understanding.^9–11^ There are hence accordingly large expectations for the future role of QSP approaches in pharmaceutical development, given their inherent mechanistic nature. For example, it is conceivable that QSP approaches may be used to accurately simulate drug efficacy and toxicity, respectively, to optimize risk-benefit ratios or for a model-based design of treatment schedules. Also, QSP models may be used for the integration and subsequent contextualization of data from all levels of biological organization, ranging from patient anthropometry to phenotypic molecular variants.

In this work, the PK/PD behavior of cyclooxygenase-2 (COX-2) and 5-lipoxygenase (5-LOX) inhibitors is investigated in a proof-of-concept study to illustrate the future potential of QSP approaches. The COX-2 and 5-LOX pathways are well-known therapeutic targets in the treatment of pain and inflammatory diseases. They represent key enzymes in the arachidonic acid metabolism and are directly involved in the production of inflammatory mediators such as prostaglandins and leukotrienes.^12^ Several anti-inflammatory drugs exert their action by blocking COX-2, which results in a decrease of prostaglandin synthesis.^13^ The inhibition of 5-LOX also seems to be a promising therapeutic strategy as it was demonstrated that leukotrienes play a major role in inflammation processes.^13^ However, the development of novel selective 5-LOX inhibitors has often failed due to efficacy or safety reasons.^14^ The only approved 5-LOX inhibitor that showed significant efficacy in the treatment of asthma is zileuton.^15^ As leukotrienes may play a role in growth and survival processes of gastrointestinal cancers, it has been suggested that zileuton should be administered concomitantly with COX-2 inhibitors used in cancer therapy to counteract leukotriene oversynthesis.^16^ In recent years, a novel class of dual COX-2/5-LOX inhibitors has emerged in the treatment of inflammatory diseases to reach optimal anti-inflammatory activity, on the one hand, and to reduce adverse side effects, such as gastrointestinal toxicity, caused by selective COX-2 inhibitors (e.g., celecoxib) or nonsteroidal anti-inflammatory drugs (e.g., diclofenac), on the other.^12,13^ Licofelone,^17^ for instance, a promising dual COX-2/5-LOX inhibitor that has successfully completed clinical phase III showed lower gastrointestinal damage in animals in comparison to anti-inflammatory drugs such as diclofenac.^18,19^

The impact of drug interactions both on drug PK and on cellular effects is next addressed. Notably, this analysis represents a prototypical question in pharmaceutical development programs. Rifampicin, a drug primarily used in tuberculosis treatment, is used here, which is often co-administered with other drugs, for instance, in HIV-infected patients because of tuberculosis coinfection. In addition to its antimycobacterial action, rifampicin also induces expression of cytochrome P450 (CYP) enzymes, particularly CYP3A4, by binding to pregnane X receptor (PXR), which further heterodimerizes with the retinoid X receptor (RXR).^20^ The formed PXR/RXR complex is finally translocated into the nucleus where it acts as transcription factor for several CYP enzymes involved in phase I metabolism of xenobiotics. As rifampicin is a potent inducer of CYPs, drug interactions may occur during co-treatment potentially reducing plasma concentrations and drug efficacy of the co-administered drugs.^20^ For instance, drug clearances of diclofenac and celecoxib has been significantly increased by rifampicin pre-treatment in healthy human volunteers most likely owing to rifampicin-induced activation of CYP enzymes.^21,22^

Taken together, a physiologically based pharmacokinetic (PBPK)/PD QSP approach is presented in this study and applied to evaluate the drug efficacy in different therapeutic situations. A selective and non-selective COX-2 inhibitor, celecoxib and diclofenac, respectively, is considered here as well as a selective 5-LOX inhibitor, zileuton, and a dual COX-2/5-LOX inhibitor, licofelone. The impact of pre-treatment with rifampicin on the drug clearances of COX-2 and 5-LOX inhibitors and, consequently, on their pharmacological action is also investigated. In the presented QSP approach, drug-specific PBPK models and existing cellular models of arachidonic acid metabolism^23^ and rifampicin-induced CYP activation^24^ are coupled. PBPK modeling is used to mechanistically assess PK of COX-2 and 5-LOX inhibitors and to simulate drug ADME at the whole-body level. The underlying model structure of PBPK models enables the quantitative simulation of drug-specific PK profiles in different organ compartments such as the interstitial or intracellular space.^25^ These PK profiles are in turn used to dynamically describe the input signal for the cellular network models. At the PD level, drug action of COX-2 and 5-LOX inhibitors is evaluated through the quantitative simulation of cellular biomarkers, i.e., prostaglandins and leukotrienes. The estimated decrease in prostaglandin formation over time for diclofenac and celecoxib is, moreover, correlated with pain relief observed in humans.^26,27^ Furthermore, the predicted increase in drug clearance provoked by rifampicin-induced activation of different CYP enzymes is compared with clinical data for celecoxib and diclofenac,^21,22^ and optimized dosing schedules are provided for co-medication to compensate for the decrease in drug action represented by the reduction of prostaglandins.

## Results

### General overview of the PBPK/PD QSP approach and its use to predict drug efficacy in humans

**Fig. 1:**
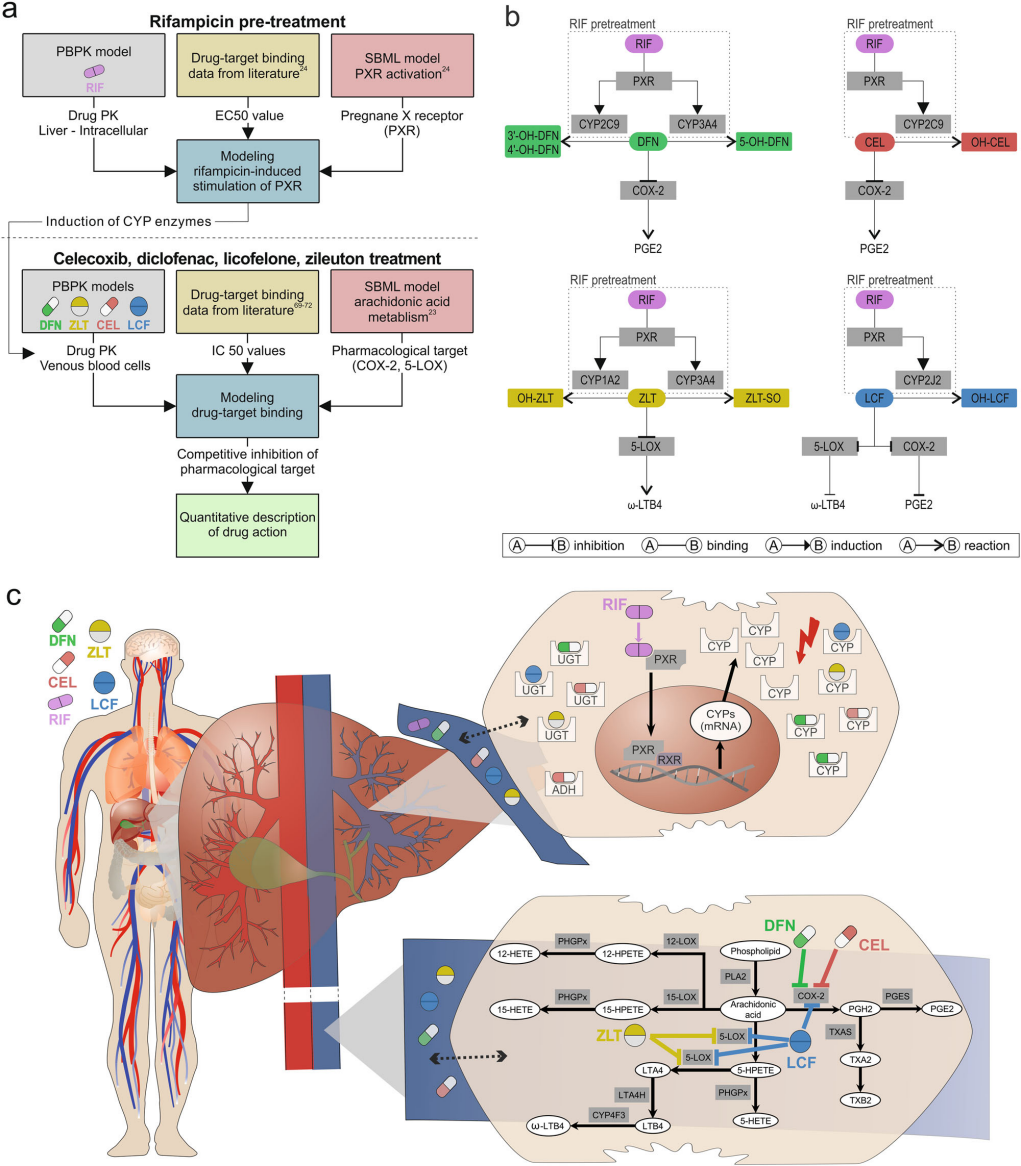
General overview of the PBPK/PD QSP approach and its use to predict drug efficacy of COX-2 and 5-LOX inhibitors in humans. The developed PBPK/PD QSP approach couples drug-specific whole-body PBPK models with computational models of arachidonic acid metabolism^23^ and rifampicin-induced CYP induction.^24^ The inhibition of COX-2 and 5-LOX and the following decrease in prostaglandin and leukotriene formation, as well as rifampicin induction of CYP enzymes and the potential impact on the pharmacokinetics and, subsequently, on the pharmacodynamics of COX-2 and 5-LOX inhibitors can be thus quantitatively described. **a** Workflow **b** Key processes involved. **c** Schematic representation. Diclofenac, DFN; hydroxy, OH; celecoxib, CEL; zileuton, ZLT; sulfoxide, SO; licofelone, LCF; rifampicin, RIF; pregnane X receptor, PXR; retinoid X receptor, RXR; leukotriene, LT; prostaglandin, PG, thromboxane, TX; hydroxyeicosatetraenoic acid, HETE; hydroperoxyeicosatetraenoic acid, HPETE

In this work, a PBPK/PD QSP approach at the whole-body level is applied using multiscale modeling (Fig. 1). PBPK/PD simulations are thereby performed to quantify drug efficacy and induction of CYP enzymes by coupling whole-body PBPK models, at the organism level, and computational models of biological processes represented in System Biology Markup Language (SBML),^28^ at the cellular level. Notably, the impact of PK drug interactions on drug efficacy can thereby also be taken into account. The PBPK/PD QSP approach is applied on different COX-2 and 5-LOX inhibitors with and without a pre-treatment with rifampicin (Fig. 1). Drug efficacy and the impact of drug interactions on the PK behavior are thereby evaluated in eleven therapeutic situations (Table 1). In this context, the following drug effects are simultaneously addressed: (i) Diclofenac and celecoxib inhibit COX-2, whereas zileuton reversibly binds to 5-LOX. In contrast, licofelone interacts with both enzymes owing to its dual-acting nature.^17^ Prostaglandin or leukotriene formation is reduced in consequence of the inhibition of COX-2 or 5-LOX. (ii) Rifampicin increases the expression of various CYP enzymes in humans, predominantly in the liver, by binding to PXR, which may subsequently results in PK drug interactions (Fig. 1).^20^

**Table 1:** Overview of the use of the PBPK/PD QSP approach

ID	Applied drug(s)	Pharmacological action	Results	Fig.
1	CEL	Single COX-2 inhibition	Drug efficacy	4a
2	DFN	Single COX-2 inhibition	Drug efficacy	4a
3	RIF + CEL	Single COX-2 inhibition	PK drug interaction and impact on drug efficacy	4a
4	RIF + DFN	Single COX-2 inhibition	PK drug interaction and impact on drug efficacy	4a
5	LCF	Dual COX-2/5-LOX inhibition	Drug efficacy	4b
6	CEL + ZLT	Single COX-2/Single 5-LOX inhibition	Drug efficacy	4b
7	DFN + ZLT	Single COX-2/Single 5-LOX inhibition	Drug efficacy	4b
8	DFN	Single COX-2 inhibition	Correlation of prostaglandin decrease and pain relief	5a
9	CEL	Single COX-2 inhibition	Correlation of prostaglandin decrease and pain relief	5b
10	RIF + DFN	Single COX-2 inhibition	Comparison of observed and predicted PK drug interaction	6a
			PK drug interaction and impact on prostaglandin formation	6d
11	RIF + CEL	Single COX-2 inhibition	Comparison of observed and predicted PK drug interaction	6a
			PK drug interaction and impact in prostaglandin formation	6d

Applications of the PBPK/PD QSP approach including the applied drugs, the pharmacological action and the main results illustrated in the correspondent figure. Celecoxib, CEL; diclofenac, DFN; licofelone, LCF; rifampicin, RIF; zileuton, ZLT; cyclooxygenase, COX; lipoxygenase, LOX

As PBPK models explicitly describe unbound drug concentrations, they may directly be coupled with computational models of biological processes, at the cellular scale, by drug–target binding in order to reflect the PD response of the considered drugs (Fig. 1). Following oral administration of therapeutically relevant doses, PK profiles of the COX-2 and 5-LOX inhibitors are simulated in venous blood cells, whereas drug concentrations of rifampicin are predicted in the intracellular space of the liver. Simulated drug concentrations are linked via reversible binding to the respective biological targets (COX-2, 5-LOX, or PXR) present in the cellular models of arachidonic acid metabolism^23^ and rifampicin-induced CYP activation.^24^ The pharmacological activity of diclofenac, celecoxib, zileuton, and licofelone is quantified by the decrease in prostaglandin and leukotriene synthesis. The potential impact of rifampicin pre-treatment on the drug action is investigated by considering PK drug interactions.

### Validation of therapeutic drug concentrations simulated using PBPK modeling

**Fig. 2:**
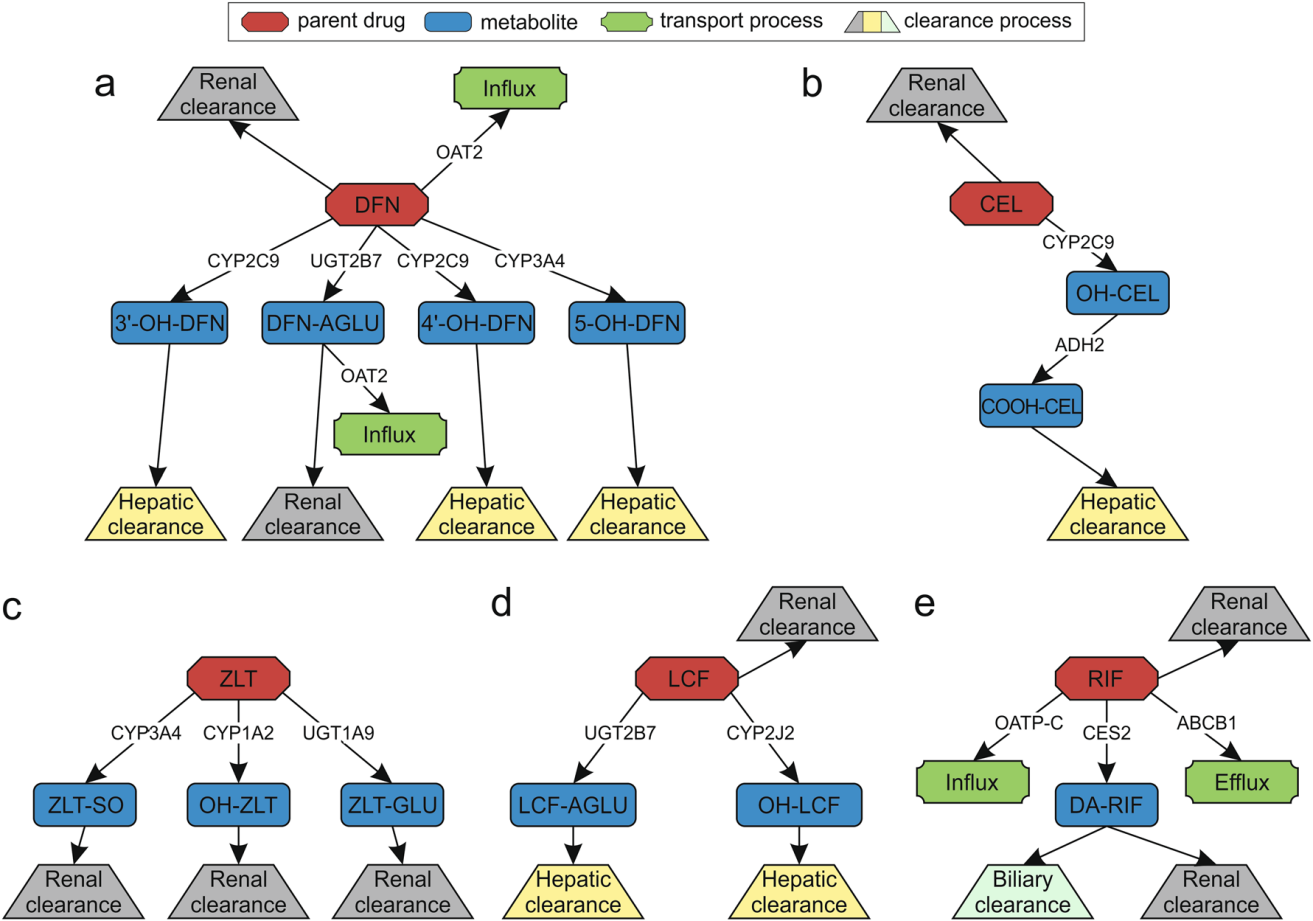
Scheme of the ADME processes implemented in the PBPK models. Thirty-two biological processes representing ADME behavior in the developed PBPK models of **a** diclofenac, **b** celecoxib, **c** zileuton, **d** licofelone, and **e** rifampicin (red; parent drugs) illustrating active drug transport (influx, efflux; green), metabolizing reactions for phase I and phase II metabolites (blue), as well as specific clearance processes (renal, hepatic, and biliary; gray, yellow, and light green). Metabolic enzymes and transporters are presented next to the respective reaction. Diclofenac, DFN; hydroxy, OH; acyl glucuronide, AGLU; celecoxib, CEL; carboxy, COOH; glucuronide, GLU; zileuton, ZLT; sulfoxide, SO; licofelone, LCF; rifampicin, RIF; desacetyl, DA

**Fig. 3:**
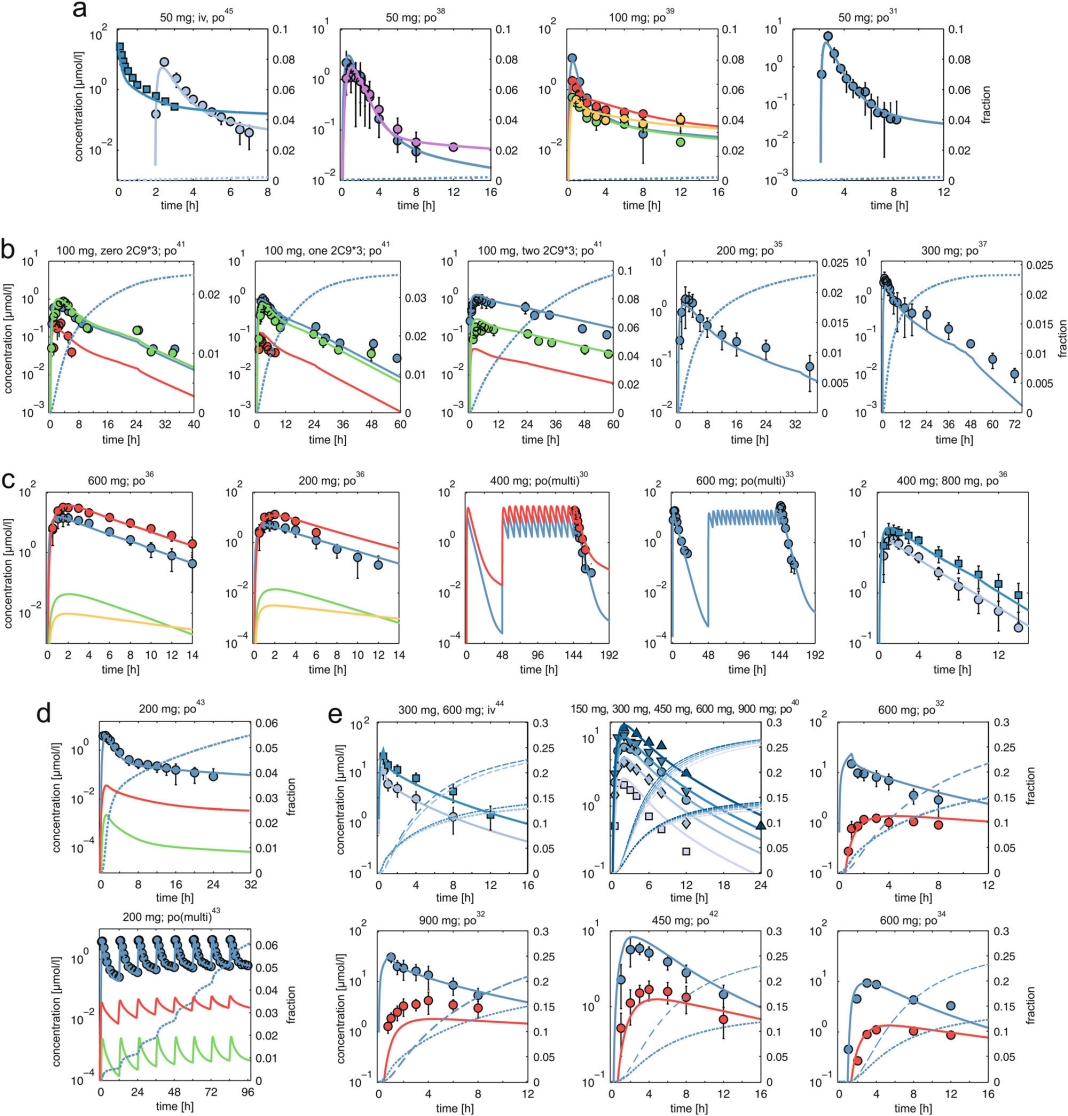
Validation of whole-body PBPK models. Simulated plasma concentration-time curves (lines) are assessed with experimental PK profiles (circles) for **a** diclofenac (DFN = blue, 4’-OH-DFN = red, 5-OH-DFN = green, 3’-OH-DFN = yellow, DFN-AGLU = purple), **b** celecoxib (CEL = blue, OH-CEL = red, COOH-CEL = green), **c** zileuton (ZLT = blue, ZLT-GLU = red, ZLT-SO = green, OH-ZLT = yellow, **d** licofelone (LCF = blue, LCF-AGLU = red, OH-LCF = green, and **e** rifampicin (RIF = blue, DA-RIF = red). Renal excretion rates are simulated for celecoxib, diclofenac, licofelone, and rifampicin (dotted blue lines). In case of rifampicin, biliary excretion rates are additionally presented (dashed blue lines). References, dose levels and administration routes (iv and po) of the experimental data are shown above each plot. In case of several administered doses displayed in the same plot, correspondent simulations are presented in ascending order from light blue to dark blue. Diclofenac, DFN; hydroxy, OH; acyl glucuronide, AGLU; celecoxib, CEL; carboxy, COOH; glucuronide, GLU; zileuton, ZLT; sulfoxide, SO; licofelone, LCF; rifampicin, RIF; desacetyl, DA; zero, one, two 2C9*3, CYP2C9 genotypes

It has been argued that a quantitative understanding of on-target drug exposure is crucial in clinical phases of pharmaceutical development programs.^29^ An accurate description of on-target drug PK is hence mandatory for generating confidence in further pharmacological simulations and predictions. In a first step, human whole-body PBPK models are therefore developed and used in the presented PBPK/PD QSP approach to simulate PK of celecoxib, diclofenac, licofelone, rifampicin, and zileuton. These drug-specific PBPK models are validated by use of clinical data from literature.^30–45^ In total, 32 ADME processes are implemented in the corresponding PBPK models to represent the ADME behavior of the considered drugs (Fig. 2). Simulations of drug plasma concentration-time profiles with the various established PBPK models show a very good agreement with clinical data measured in humans (Fig. 3, and Supplementary Fig. S1).^30–45^ Notably, the validated PBPK models can be used for predicting accurate PK profiles in different organ compartments (e.g., intracellular space of the liver) owing to the underlying model structure representing a large level of physiological detail.

In a subsequent step, therapeutic drug concentrations are simulated and used as input to quantify the pharmacological activity of the considered COX-2 and 5-LOX inhibitors.

### Evaluation of drug efficacy predicted for COX-2 and 5-LOX inhibitors in therapeutic situations

To quantitatively evaluate the drug efficacy of celecoxib, diclofenac, licofelone, and zileuton, the established PBPK/PD QSP approach is applied (Fig. 1). After administration of therapeutic doses, the drug efficacy of the COX-2 and 5-LOX inhibitors are calculated for a duration of 6 h by comparing the decrease in prostaglandin and leukotriene formation, respectively, to the drug-free treatment. Only COX-2 inhibition of the non-selective COX inhibitor diclofenac was here considered as, at first, diclofenac shows a higher selectivity on COX-2 than COX-1,^46–50^ and, second, COX-1 was not considered in the existing model of arachidonic acid metabolism.^23^

Rifampicin is administered concomitantly with COX-2 inhibitors in several clinical applications and may decrease drug plasma concentrations owing to an increased metabolic activity of CYP enzymes.^21,22^ To illustrate the application of QSP approaches in the analysis of drug interactions, the effect of pre-administered rifampicin, the perpetrator drug, on the PD response of celecoxib and diclofenac, the victim drugs, is also investigated here (Table 1). It has been estimated that rifampicin is only a weak inducer of CYP1A2 and 2J2 (see Methods), which are both involved in the metabolism of licofelone and zileuton (Fig. 2). Furthermore, rifampicin-induced activation of CYP3A4 (Supplementary Fig. S2) has no significant effect on zileuton concentrations as zileuton-sulfoxide, which is formed by CYP3A4 from the parent drug (Fig. 2), contributes little to the overall metabolism of zileuton. Thus, no significant alterations in licofelone and zileuton concentrations induced by rifampicin are observed and a potential pre-treatment with rifampicin is not analyzed any further.

The drug efficacy of the dual COX-2/5-LOX inhibitor licofelone is compared with a potential co-treatment of a COX-2 inhibitor (celecoxib or diclofenac) and the 5-LOX inhibitor zileuton (Table 1). As no drug interactions between celecoxib or diclofenac combined with zileuton were found (www.drugs.com/drug_interactions.html, 2017), it is assumed that the PD response of both is not affected during co-administration with zileuton.

**Fig. 4:**
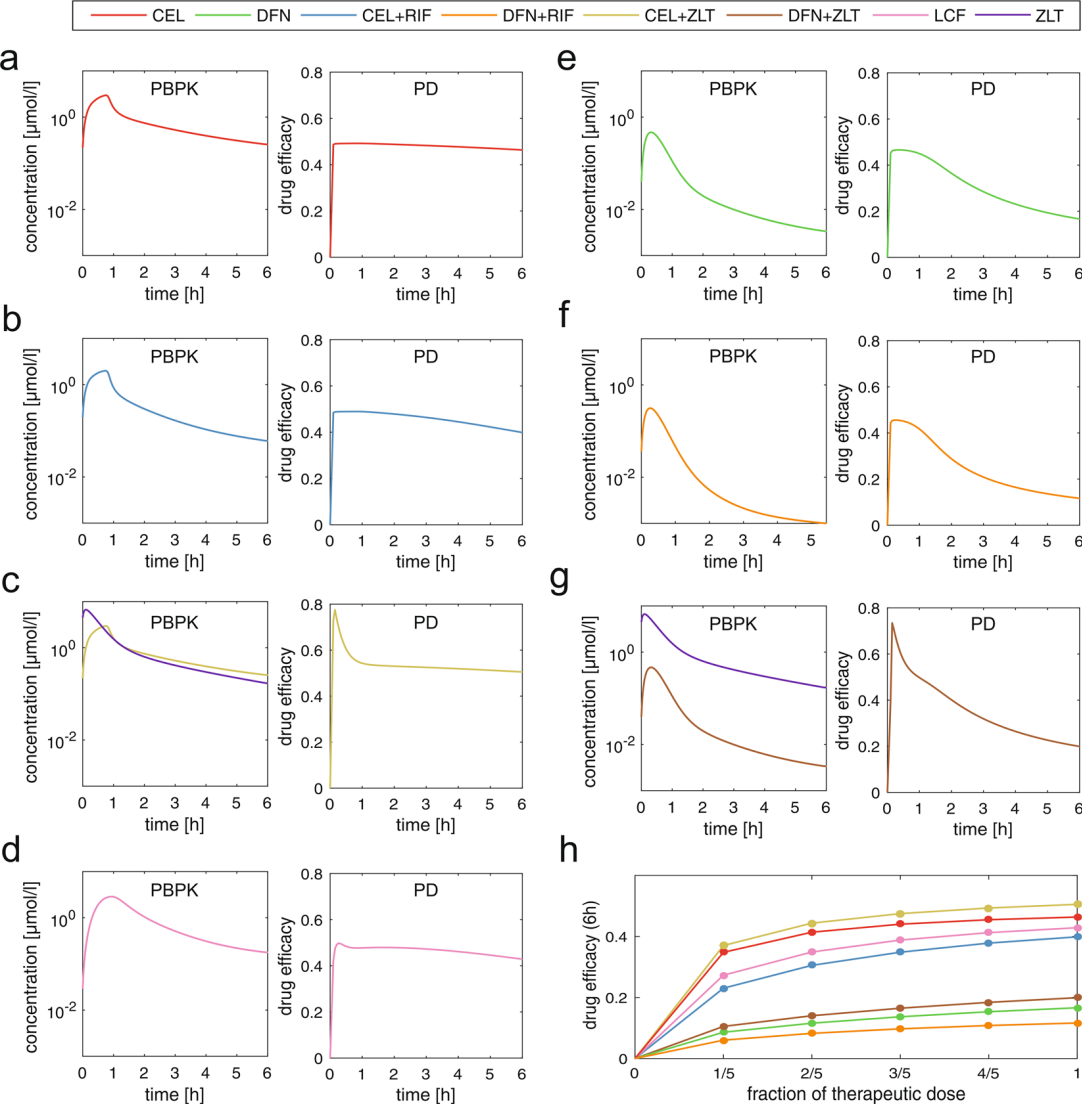
PBPK/PD simulations of COX-2 and 5-LOX inhibitors in different therapeutic situations. Drug concentrations in venous blood cells and drug efficacy expressed as drug efficacy score following oral administration of therapeutic doses (CEL: 100 mg; DFN: 50 mg; LCF: 200 mg; ZLT: 600 mg; pre-treatment with RIF: 600 mg q.d. over 1 week) are predicted for a duration of 6 h. **a** CEL **b** CEL **+** RIF **c** CEL + ZLT **d** LCF **e** DFN **f** DFN + RIF **g** DFN + CEL. **h** Dose escalation studies monitoring drug efficacy for stepwise increasing the dose level until the therapeutic dose is reached. Celecoxib, CEL; diclofenac, DFN; licofelone, LCF; rifampicin, RIF; zileuton, ZLT

Considering administration of single inhibitors, celecoxib shows highest drug efficacy with nearly unchanged behavior after 6 h probably owing to its long half-life (drug efficacy score DES_CEL, 6 h_ = 0.46) (Fig. 4a).^51^ In contrast, diclofenac reaches lower drug efficacy with an obvious decrease after 6 h because of its short residence time within the body (DES_DFN, 6 h_ = 0.17) (Fig. 4e).^51^ A pre-administration of rifampicin demonstrates a moderate effect on the PD response of celecoxib (DES_CEL+RIF, 6 h_ = 0.39, DES_CEL, 6 h_ = 0.46) and diclofenac (DES_DFN+RIF, 6 h_ = 0.11, DES_DFN, 6 h_ = 0.17) (Fig. 4a, b, e, f and Supplementary Fig. S2).

In case of inhibiting both, prostaglandin and leukotriene formation by reversibly blocking COX-2 and 5-LOX, an additional effect is observed for celecoxib and diclofenac co-applied with zileuton after 6 h (DES_CEL+ZLT, 6 h_ = 0.51, DES_CEL, 6 h_ = 0.46; DES_DFN+ZLT, 6 h_ = 0.20, DES_DFN, 6 h_ = 0.17) (Fig. 4a, c, e, g). This additional effect is found to be most prominent in the early phase after administration (DES_CEL+ZLT, 0.5 h_ = 0.61, DES_CEL, 0.5 h_ = 0.49; DES_DFN+ZLT, 0.5 h_ = 0.57, DES_DFN, 0.5 h_ = 0.46) (Fig. 4a, c, e, g). Licofelone shows slightly lower drug efficacy compared to co-treatment of celecoxib and zileuton, whereas it even outperforms a co-administration of diclofenac with zileuton after 6 h (DES_LCF, 6 h_ = 0.43, DES_CEL+ZLT, 6 h_ = 0.51, DES_DFN+ZLT, 6 h_ = 0.20) (Fig. 4c, d, g).

To evaluate the increase in drug efficacy for different doses, dose escalation studies are finally performed for the different treatment scenarios (Fig. 4h). To this end, the dose level is stepwise increased until the therapeutic dose is reached. The respective drug efficacy after 6 h is calculated simultaneously to monitor the pharmacological action over the wide range of applied dose levels.

In the following, the pharmacological activity as estimated from inflammatory mediators is correlated with clinical outcome data on the example of celecoxib and diclofenac.

### Correlation of predicted decrease in prostaglandin formation with pain relief

A major therapeutic action of COX-2 inhibitors is to treat acute inflammatory or post-traumatic pain through their analgesic effect. To validate biomarker-based estimations of pharmacological activity of celecoxib and diclofenac with diagnosable clinical outcome data, pain relief scores over time are correlated with the predicted decrease in prostaglandin formation used to represent a quantitative measurement for drug action.^26,27^

In comparison with the clinical study of diclofenac,^27^ key PK parameters could be accurately reproduced by our PBPK model using a dissolved formulation for 25 mg and 50 mg diclofenac (25 mg DFN: Cmax_sim_ = 788, Cmax_exp_ = 1125 ± 486; Tmax_sim_ = 0.40, Tmax_exp_ = 0.45 ± 0.13; AUC_sim_ = 724, AUC_exp_ 603 ± 163; and 50 mg DFN: Cmax_sim_ = 1609, Cmax_exp_ = 2035 ± 725; Tmax_sim_ = 0.40; Tmax_exp_ = 0.48 ± 0.20, AUC_sim_ = 1491, AUC_exp_ 1232 ± 296; AUC(0→∞), ng*h/ml; Cmax, ng/ml; Tmax, h).^52^ Hence, the decrease in prostaglandin formation is determined in the following by using the dissolved formulation.

**Fig. 5:**
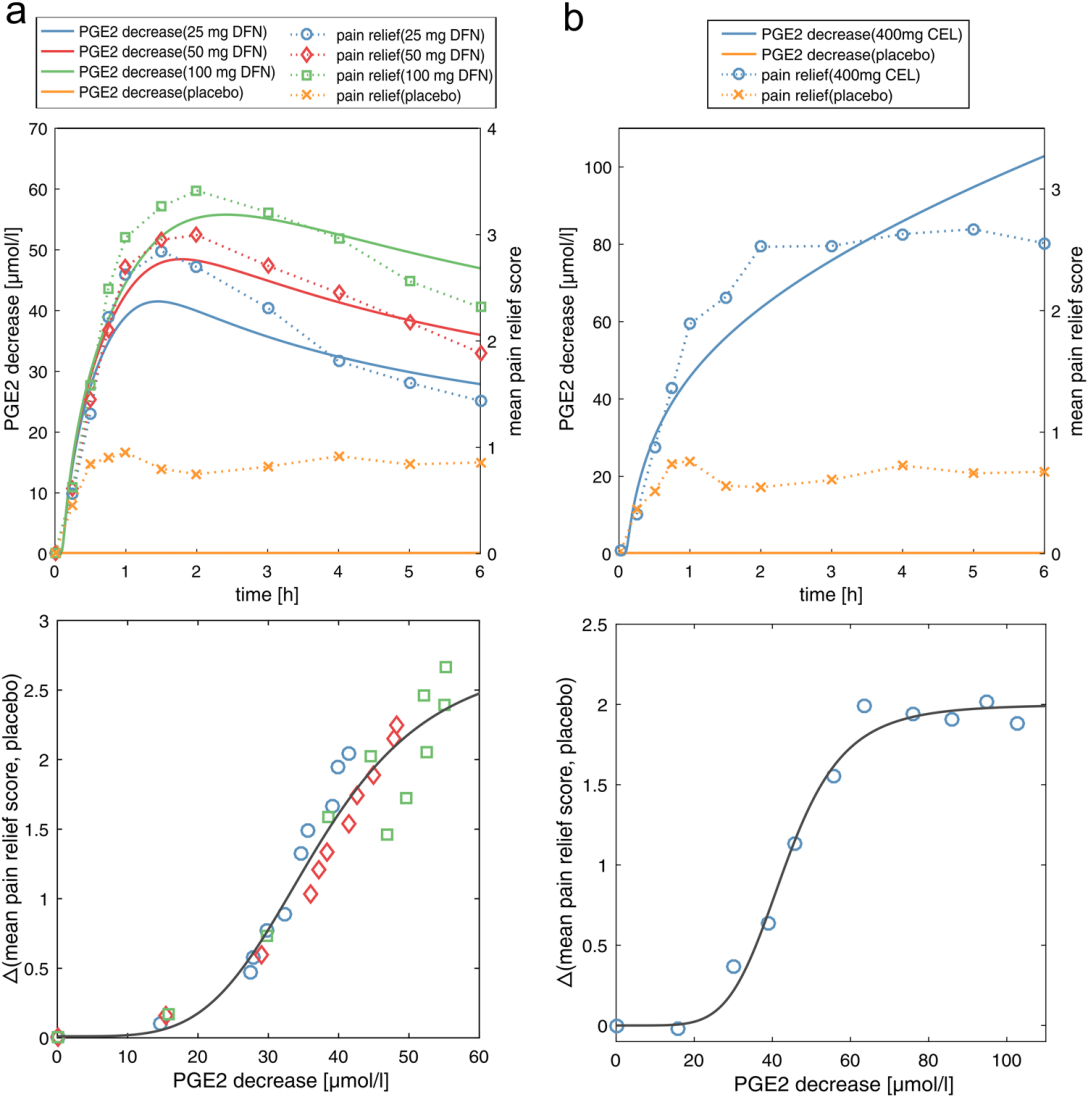
Correlation of PGE2 decrease with pain relief. Predicted decrease in PGE2 formation over time compared with the drug-free treatment is correlated with mean pain relief scores observed in patients after 6 h for **a** 25 mg, 50 mg, and 100 mg diclofenac, and **b** 400 mg celecoxib. Placebo effects of both studies are additionally shown. Celecoxib, CEL; diclofenac, DFN; prostaglandin E2, PGE2

Strong correlation results are found for both, diclofenac and celecoxib (Fig. 5a, b, Supplementary Table S4). The model with the best fit, which turned out to be the Hill equation ((a*x^b^)/(c^b^ + x^b^); DFN: a = 2.8 CI (2.0, 3.5), b = 4.4 CI (2.6, 6.3), c = 37.2 CI (31.9, 42.5); CEL: a = 2 CI (1.9, 2.1), b = 5.7 CI (3.7, 7.8), c = 43.4 CI (40.6, 46.1); CI = 95% confidence interval), was selected based on different quality measures (Supplementary Table S4). These findings indicate a strong relation between the predicted PGE2 decrease and the observed pain relief of celecoxib and diclofenac.

In the following conclusive analysis, the impact of rifampicin on the therapeutic action of celecoxib and diclofenac is studied in more detail at the PK and PD level and is, furthermore, validated with clinical observations in humans.

### PBPK/PD analysis of celecoxib and diclofenac administration pre-treated with rifampicin

**Fig. 6:**
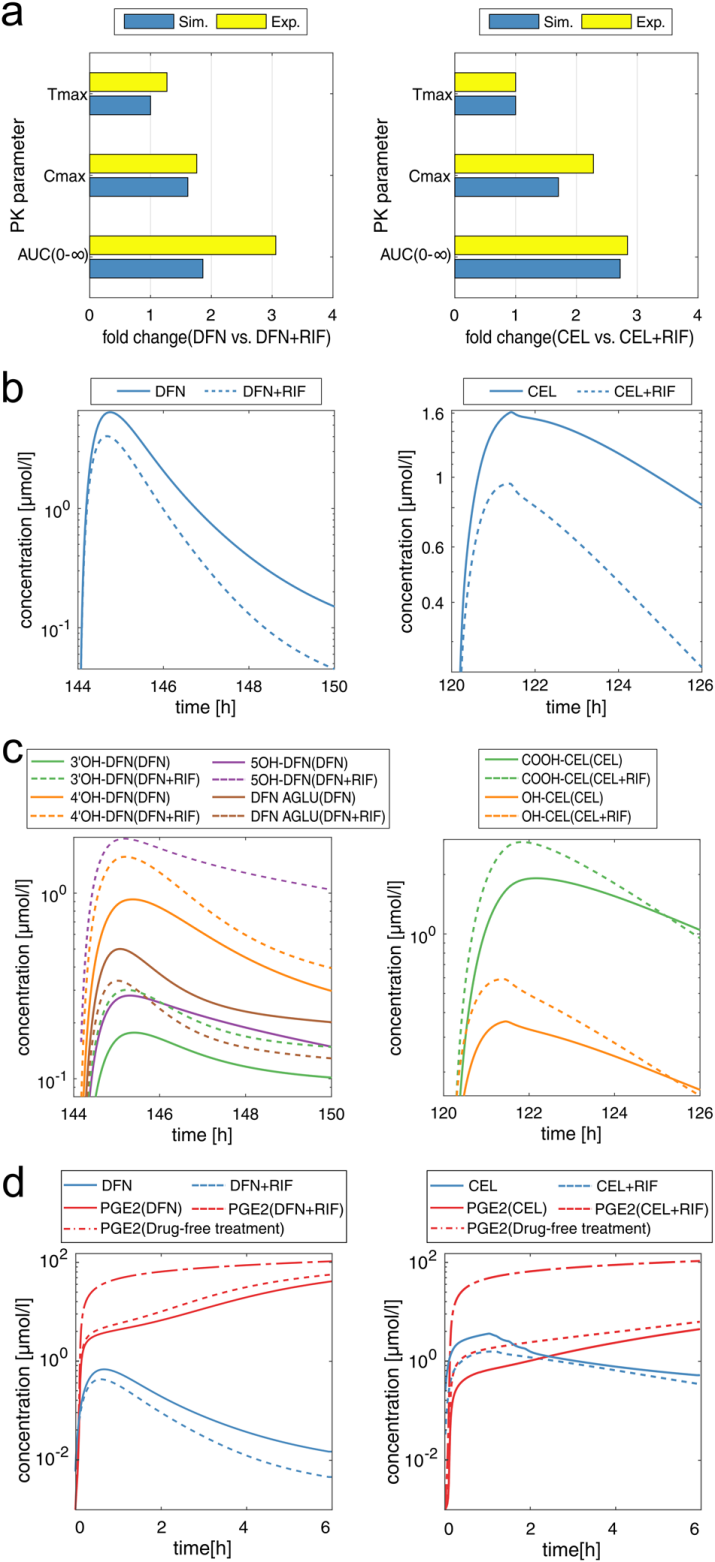
PBPK/PD analysis of celecoxib and diclofenac pre-treated with rifampicin. PBPK/PD analysis of COX-2 inhibitors following oral administration of 200 mg celecoxib and 100 mg diclofenac with and without a pre-treatment with 600 mg and 450 mg rifampicin q.d. over 5 and 6 days, respectively. **a** Comparison of key PK parameters between simulated and experimental data. **b** Simulation of venous blood plasma concentrations in humans **c** Metabolite analysis showing venous blood plasma concentrations of correspondent metabolites. **d** PBPK/PD simulations reflecting drug concentrations of celecoxib and diclofenac in venous blood cells, as well as induced changes in PGE2 formation over time. Celecoxib, CEL; diclofenac, DFN; hydroxy, OH; acyl glucuronide, AGLU; carboxy, COOH; rifampicin, RIF; prostaglandin E2, PGE2; once daily, “q.d”

Rifampicin-induced stimulation of PXR may change the activity of crucial phase I enzymes, such as CYP3A4 or 2C9. As these enzymes are involved in the clearance of celecoxib and diclofenac from the human body (Fig. 2),^51^ their altered pharmacological activities caused by a PK drug interaction with rifampicin are here investigated for two clinical studies, in which 200 mg celecoxib and 100 mg diclofenac are orally applied with a pre-treatment with 600 mg and 450 mg rifampicin q.d. (once daily) over 5 and 6 days, respectively.^21,22^ Alterations in the concentration-time profiles of affected metabolites are analyzed and changes in key PK parameters of the parent drugs are validated using clinical data (Fig. 6). The effect of rifampicin pre-treatment is also explored at the PD level thereby quantitatively evaluating the diminished drug efficacy provoked by the PK drug interaction. Optimized dose levels are finally suggested to reach the same therapeutic efficacy of co-medication as for single administration.

The increased activity of CYP3A4 and 2C9 induced by pre-treatment with rifampicin leads to a significant increase in drug clearance of celecoxib and diclofenac particularly during first-pass metabolism (Fig. 6b). The changes in the PK of celecoxib and diclofenac for co-administration with rifampicin are in accordance with experimental observations demonstrated by small deviations in key PK parameters (Cmax, Tmax, and AUC) between the simulated and the experimental data (Fig. 6a). These results present a validation of the predicted PK drug interactions between the victim drugs, celecoxib and diclofenac, and the perpetrator drug, rifampicin, by use of clinical data obtained in healthy volunteers.^21,22^

A metabolite analysis is next performed to get quantitative insights into temporal changes in plasma concentration of celecoxib and diclofenac metabolites. Rifampicin-induced activation of CYPs has a significant impact on 3'-hydroxy-diclofenac, 4'-hydroxy-diclofenac, and 5'-hydroxy-diclofenac reflected by an increase in their plasma concentrations (Fig. 6c). In case of celecoxib, phase I and phase II metabolites, hydroxy-celecoxib and carboxy-celecoxib, respectively, are both affected by rifampicin indicated by considerable alterations in their PK profiles (Fig. 6c).

Finally, the extent of the PK drug interactions induced by rifampicin on the drug efficacy of celecoxib and diclofenac is studied at the PD level. A moderate and major reduction of their pharmacological activity is observed for both celecoxib and diclofenac, respectively, (DES_CEL+RIF, 6 h_ = 0.47; DES_CEL, 6 h_ = 0.48; DES_DFN+RIF, 6 h_ = 0.23; DES_DFN, 6 h_ = 0.30), which requires dose adjustments during co-administration with rifampicin (Fig. 6d). Dose adjustments of 2.5-fold and 1.5-fold times the initial dose are estimated for diclofenac and celecoxib, respectively, to obtain similar initial therapeutic efficacy as predicted for single drug administration (Fig. 6d).

## Discussion

In this study, a PBPK/PD QSP approach is applied to quantify drug efficacy over time for a set of COX-2 and 5-LOX inhibitors in humans (Fig. 1, Table 1). Furthermore, the impact of pre-treatment with rifampicin on their pharmacological activity is evaluated, thereby reflecting clinically relevant situations. The key step of the approach involves the linkage of whole-body PBPK models of anti-inflammatory drugs and rifampicin (Figs. 2, and 3) with cellular network models of arachidonic acid metabolism^23^ and, simultaneously, with PXR-mediated CYP induction,^24^ respectively. In brief, using this PBPK/PD QSP approach allows a quantitative description of drug-induced modulations on biological systems associated with pharmacological action following oral administration of therapeutic doses at patient level. To couple simulated PK profiles of celecoxib, diclofenac, licofelone, and zileuton with the SBML model of arachidonic acid metabolism,^23^ drug-specific IC_50_ values measured for COX-2 and 5-LOX inhibition are utilized to estimate binding affinities for competitive inhibition using the IC_50_-to-Ki converter.^53^ This simple coupling step provides the opportunity for linking various cellular network models with drug concentration-time profiles to gain more insights into the dynamics of modulated biological processes induced by drug–target binding.

The dynamic network of arachidonic acid metabolism was previously constructed in human polymorphonuclear leukocytes.^23^ As in the modeling structure, no subcompartments such as leukocytes, erythrocytes, or thrombocytes exist, and no drug concentrations measured in human polymorphonuclear leukocytes were available for validation purposes, drug PK of celecoxib, diclofenac, licofelone, and zileuton are predicted in venous blood cells as such representing a reasonable compartment during the coupling step of the PBPK/PD QSP approach. Besides its antimycobacterial activity, rifampicin acts in humans by increasing the activity of several CYP enzymes initially through binding to PXR.^20^ This secondary drug effect is modeled to evaluate the impact of co-administration of rifampicin as it is frequently applied in different clinical studies.^20–22^ Rifampicin concentrations are therefore calculated in the intracellular space of the liver because binding to PXR mainly takes place inside liver cells and the correspondent SBML model was validated before using in vitro data measured in human hepatocytes.^24^ In all cases, unbound drug concentrations are used for simulations and subsequent calculations as the fraction of the plasma protein-bound drug is generally not pharmacological active.

In addition to the findings presented in this study, the PBPK/PD QSP approach might be expanded to quantify not only drug efficacy but also to explore unwanted side effects. This, however, assumes that relevant biomarkers of potential adverse events are contained in the observed cellular network model. The adverse event of a drug may then be explored at the whole-body level, which may improve the understanding of toxic mechanisms for approved drugs or may help to assess the toxic potential of investigational drugs during drug development (Supplementary Table S1).

As PBPK modeling can also be applied to predict drug concentrations in different animals,^54^ the PBPK/PD QSP approach may be used for mice, rats, dogs, or monkeys in the preclinical phase (Supplementary Table S1). A potential usage of the in silico-based approach to predict efficacious and safe dose levels might reduce animal testing during drug discovery. This, however, requires the development of an animal PBPK model based on in vitro data (e.g., plasma protein binding) and on predicted physicochemical drug properties. Having quantitative knowledge about the influence of different dose levels on the drug efficacy of novel drugs may help to plan appropriate dosing schedules or to improve existing ones in clinical phases of drug development. Hence, PBPK/PD QSP approaches may be applied to support multiple phases in pharmaceutical development programs (Supplementary Table S1). Recently, for instance, the usage of QSP has even entered the regulatory domain. Clinical pharmacologists of the Food and Drug Administration (FDA) in the United States have utilized a QSP model to evaluate the appropriateness of a suggested dosing schedule for denosumab, a human monoclonal antibody developed for the treatment of osteoporosis.^55^

The underlying structure of PBPK models allows the simulation of reliable PK profiles in several compartments within the human body (e.g., the intracellular space of the liver) that indicates the major benefit of using PBPK modeling in this study.^56^ The presented PBPK/PD QSP approach that utilizes PBPK modeling is, thus, applicable for drugs with diverse sites of action such as the heart or the kidney assuming, though, an adequate cellular network model including the pharmacological target that is mostly responsible for the respective mode of action.

The inhibition of inflammatory mediators, such as prostaglandins and leukotrienes, is estimated by modeling the reversible binding of the considered drugs to COX-2 and/or 5-LOX. As both are pharmacological targets within the body, therapeutic effects can be quantitatively described (Fig. 4). In comparison with celecoxib, diclofenac represents a non-selective COX-2 inhibitor, which also shows an inhibitory effect of COX-1, contributing to its overall drug efficacy. The presented results simulating diclofenac efficacy and relating it to pain relief were nevertheless based solely on COX-2 inhibition as diclofenac shows a higher selectivity towards COX-2 (3–29-fold higher selectivity for COX-2 compared with COX-1),^46–50^ on the one hand. Also, it has been proposed that COX-2 is substantially involved in inflammatory processes and the pain associated with it.^13^ Furthermore, the resulting impact of prostaglandin production was here represented as decrease in PGE2 that is known to contribute to inflammatory pain.^57^ It has been demonstrated, for instance, that changes in PGE2 concentrations could be significantly correlated with headache and muscle pain in humans.^58^

Elevated levels of physiological concentrations of immunoreactive PGE2 could also be detected by implanted microdialysis probes and correlated with the onset of postoperative pain in patients.^59^ Moreover, it was shown that inhibition of PGE2 might be used to predict and identify efficacious doses for selective and non-selective COX inhibitors in humans.^50^ In addition, considering COX-1 inhibition by diclofenac would also require a suitable and validated cellular model of the arachidonic acid metabolism considering both isoforms, which was not available. The PD response of licofelone mediated by dually inhibiting COX-2 and 5-LOX can also be investigated through the use of the established PBPK/PD QSP approach. Polypharmacology is thereby covered by the consideration of multiple drug–target interactions on a single pathway.^60^ To compare the dual effect of licofelone on prostaglandin and leukotriene production, a potential co-medication of zileuton with either celecoxib or diclofenac is applied at therapeutic doses (Fig. 4). Such a combination therapy may have clinical relevance as it was suggested to co-administrate zileuton with COX-2 inhibitors in cancer therapy in order to control leukotriene oversynthesis.^16^

The predicted decrease in prostaglandin production over time induced by celecoxib and diclofenac is compared with the degree of pain relief achieved in patients after dental surgery.^26,27^ A potential increase in local inflammatory mediators after surgery can be here expected. Although the arachidonic acid metabolism network was developed in centrally obtained polymorphonuclear leukocytes, it is nevertheless assumed that utilizing this cellular network in the QSP PBPK/PD approach is representative to compare the simulated decrease in inflammatory mediators with the observed pain relief. Strong correlations are found for both drugs (Fig. 5, Supplementary Table S4), which implies the relation to clinical outcome after drug intake of therapeutic doses. Correlating pain relief scores with drug efficacy of celecoxib after 24 h showed lower but still strong correlation (Supplementary Table S4). In case of licofelone and zileuton, no adequate time-resolved clinical data were available such that no validation of the estimated therapeutic effects could be performed.

Although rifampicin-induced CYP induction is highest for CYP3A4, other CYP enzymes, such as CYP2C8 or 2C9, may be affected.^20^ The existing model for determining rifampicin-induced activation dynamics of CYP3A4^24^ is, hence, expanded for other CYP enzymes involved in the metabolism of the considered COX-2 and 5-LOX inhibitors (Fig. 2). For this, rifampicin-induced induction ratios of CYP2C9, 1A2, and 2J2 are derived with PICD (PBPK-based in vivo contextualization of in vitro toxicity data), which was recently applied to model drug interactions in humans.^61,62^ The estimated induction ratios appear to be consistent with other induction ratios observed in literature^20,63,64^ such that PK drug interactions induced by co-administration of rifampicin can be predicted even though no calibration of the expanded model with in vitro data was performed for the relevant CYP enzymes. This approach is, moreover, encouraged by predicted PK changes of celecoxib and diclofenac after co-medication with rifampicin that are in line with clinical observations (Fig. 6).^21,22^ It is worth mentioning that the altered pharmacological activity resulting from the predicted increase in drug clearance as well as the suggested dose adjustments identified consequently to recover initial therapeutic efficacy demonstrate meaningful clinical benefit. This is also in line with the FDA guidance where it was suggested to use model-based predictions in terms of evaluating the results of drug interaction studies in drug development.^65^ The effect of rifampicin on the PK of zileuton and licofelone are found to be negligible such that insignificant changes in their drug clearance are observed. Interestingly, the proposed increase in the metabolism of zileuton when combined with rifampicin^51^ could not be confirmed as the contribution of zileuton-sulfoxide, which is formed by CYP3A4, to the overall metabolism of zileuton is estimated to be quite low (Fig. 2). This can be pointed out by a metabolite analysis enabled through the use of PBPK modeling similar to what was done for diclofenac and celecoxib (Fig. 6c).^56^

In conclusion, the established PBPK/PD QSP approach presents a multiscale approach that couples whole-body PBPK models, at the organism level, with SBML models representing crucial biological processes, at the cellular level. Drug-induced modulation on cellular networks can be thus analyzed through complex PBPK/PD simulations thereby quantifying the drug efficacy of COX-2 and 5-LOX inhibitors following administration of therapeutic doses in humans. Notably, therapeutic effects of drugs dynamically interacting with multiple pharmacological targets within a biological system can be studied as well. Furthermore, PK drug interactions caused by CYP induction can be predicted and the impact on the pharmacological activity of co-administered drugs is assessable. The high modularity of the PBPK/PD QSP approach enables a generic application for different species, various drugs, and several computational models representing pharmacologically- or toxicologically relevant biological networks. It is thus conceivable to apply this approach to support various phases of the drug development process (Supplementary Table S1). The presented findings reveal quantitative insights toward a systems-level understanding of the drug action of celecoxib, diclofenac, licofelone, and zileuton in different therapeutic situations in view of assessing the effects of varying oral dose levels, predicted PK drug interactions with rifampicin, and potential combination therapies. As demonstrated in this study, using QSP clearly provides quantitative insights of therapeutic drug action in different clinical scenarios. The high extensibility of the presented PBPK/PD QSP approach may also allow the investigation of a wide range of drugs acting on different site of actions and their effect on cellular models reflecting processes that may be relevant for pharmacological or toxicological considerations. Also, the integration of cellular PD models into whole-body PBPK models represents a prototypical scenario for the portability of literature models in QSP as such supporting translational concepts in pharmaceutical development. Hence, the systematic use of QSP approaches has the clear potential to significantly support the mechanistic understanding of physiological processes governing drug ADME and drug action as well as their mutual interplay across different levels of biological organization. QSP can thus be expected to improve the rational design of more effective and less toxic drugs in the future.

## Methods

### Implementation of the PBPK/PD QSP approach

The presented PBPK/PD QSP approach is implemented in MATLAB (8.6.0; The MathWorks, Inc., Natick, MA). PBPK/PD simulations are performed by use of the MoBi® Toolbox for MATLAB (version 2.3; Bayer Technology Services GmbH) and the IQM Toolbox (version 1.0; IntiQuan GmbH, Basel; http://www.intiquan.com/iqm-tools)^66^ by coupling in-house developed PBPK models (Fig. 3) with existing SBML models published in literature (Supplementary Fig. S3).^23,24^ Initial parametrization of the SBML models were used unchanged. Only PBPK-based concentration-time profiles as well as drug-specific binding affinity values were used as input to perform PBPK/PD simulations.

### Whole-body PBPK models

Human PBPK models are established by use of the software PK-Sim® (version 6.3) that is freely available (https://github.com/Open-Systems-Pharmacology).^56^ A description of the model development process including all relevant information that are needed to reproduce the PBPK models is outlined in detail in Supplementary Information (Supplementary Table S2, Supplementary Table S3).

### Computational models of biological processes

The SBML model of enzyme induction represents the binding of rifampicin to PXR in human liver cells and the further increase in CYP3A4 mRNA expression levels as well as CYP3A4 enzyme activity.^24^ The SBML model of the complex arachidonic acid metabolism network consists of several reactions describing different degradation pathways of arachidonic acid in human polymorphonuclear leukocytes obtained from venous blood.^23^ Twenty-four positive and negative feedback loops are additionally incorporated to represent the regulatory level of inflammatory mediators. Both SBML models from literature were established by fitting to specific experimental data.^23,24^

### Simulation of therapeutic drug PK

Drug concentration-time profiles are simulated following oral administration of therapeutic doses applied in clinical practice. As individual, a European man with a weight of 73 kg and an age of 30 years was used. It was assumed that the drug is in dissolved form. Therapeutic dose levels (CEL: 100 mg; DFN: 50 mg; LCF: 200 mg; and ZLT: 600 mg) are taken from Drugs.com (https://www.drugs.com/) and from literature.^30–45^

### Modeling pharmacokinetic drug interactions between rifampicin and COX-2 and 5-LOX inhibitors

Increased CYP3A4 activity induced by rifampicin is predicted according to Yamashita et al.^24^ using as input own simulations of intracellular rifampicin concentrations and an EC50 of 1.18 µM for binding to PXR (Supplementary Fig. S2). Oral dosing of 600 mg rifampicin q.d. over one week is applied as it reflects common pre-treatment.^24^ In the PBPK models of the COX-2 and 5-LOX inhibitors, the correspondent catalytic rate constants (kcat) are adjusted with respect to the predicted increase in CYP3A4 activity. As tissue-specific gene expression data are used to estimate abundances of ADME proteins in different compartments, adjusting kcat values may also influence the extrahepatic metabolism.^67^ In order to consider rifampicin-stimulation of other CYP enzymes than CYP3A4 involved in the metabolism of the victim drugs (CYP2C9, 2J2, and 1A2), a previously developed multiscale approach called PICD is used.^61^ PICD allows estimating drug-induced changes at the transcriptional level for different enzymes. PICD-based predictions are generated for an oral dose of 600 mg rifampicin to obtain respective induction ratios relative to CYP3A4 (CYP2C9: 0.18; CYP2J2: 0.03; CYP1A2: 0.02). These induction ratios are finally applied to estimate rifampicin-induced activation of CYP2C9, 2J2, and 1A2 analogously to CYP3A4 activation.

### Modeling pharmacodynamic responses of COX-2 and 5-LOX inhibitors

Drug efficacy of COX-2 and 5-LOX inhibitors is estimated by comparing inflammatory mediators to the drug-free treatment.^23^ The SBML model of arachidonic acid metabolism is adapted accordingly assuming Michaeli–Menten kinetics and competitive inhibition as described in Yang et al.^23^ A residence time of 5 mins in the oral cavity is assumed for the drugs meaning that the beginning of inhibitory effects starts after that time.^68^ Binding affinity (Ki) to the pharmacological targets (Ki_CEL,COX-2_ = 0.017; Ki_DFN,COX-2_ = 0.018; Ki_LCF,COX-2_ = 0.032; Ki_LCF,5-LOX_ = 0.056; Ki_ZLT,5-LOX_ = 0.021) are determined by using the IC_50_-to-Ki converter^53^ with IC_50_ values taken from literature as input.^69–72^ It was assumed that inhibition of COX-2 and 5-LOX was not saturated. To quantify the efficacy of a drug *d* at a given time *t* (in minutes), a drug efficacy score (DES) is computed as follows:

1 $${\mathrm{DES}}_{d,t} = \frac{{\frac{{PGE2_t^{control} - PGE2_t^d}}{{PGE2_t^{control}}} + \frac{{{\mathrm{\omega }} LTB4_t^{control} - {\mathrm{\omega }} LTB4_t^d}}{{{\mathrm{\omega }} LTB4_t^{control}}}}}{2}$$

where PGE2^control^, ωLTB4^control^, PGE2^d^, and ωLTB4^d^ are the concentrations of the affected inflammatory mediators for the drug-free scenario and the drug-treated case, respectively. PGE2 and ωLTB4 were used as inflammatory mediators to represent changes in prostaglandin and leukotriene formation.

### Correlation of predicted decrease in prostaglandin formation with pain relief

PGE2 decrease expressed as difference of PGE2 concentrations between the drug-free treatment and the drug-treated case are correlated with the difference between mean pain relief scores after 6 h and the respective placebo effects.^26,27^ For both drugs, different models were tested and the best fit (sigmoidal hill equation) was chosen according to several model quality measures (Supplementary Table S4). The oral dose levels of diclofenac were 25 mg, 50 mg, and 100 mg, whereas the applied dose of celecoxib was 400 mg.^26,27^ Postoperative pain relief was observed in patients after oral dental surgery. Fifty-seven patients were treated with celecoxib, whereas 197 patients were treated with different doses of diclofenac (25 mg: *n* = 63, 50 mg, *n* = 68, 100 mg = 66).

### Data availability

The SBML models of arachidonic acid metabolism and rifampicin-induced CYP3A4 activation can be found in literature.^23,24^ All information that are needed to reproduce the PBPK models are provided in Supplementary Information (Supplementary Tables S1 and S2) and in literature.^30–45^ Further data are available upon request.

## Electronic supplementary material

Supplementary Information

Supplementary Table S1

Supplementary Table S2

Supplementary Table S3

Supplementary Table S4

Supplementary Fig. S1

Supplementary Fig. S2

Supplementary Fig. S3

## Electronic supplementary material

**Supplementary information** accompanies the paper on the *npj Systems Biology and Applications* website (10.1038/s41540-018-0062-3).
