# Supplementary material for: Using quantitative systems pharmacology to evaluate the drug efficacy of COX-2 and 5-LOX inhibitors in therapeutic situations
Source: NPJ Syst Biol Appl. 2018 Aug 3;4:28. doi: 10.1038/s41540-018-0062-3 (PMC6072773; doi:10.1038/s41540-018-0062-3)
Supplement: Supplementary file 1 — Supplementary Information [file 41540_2018_62_MOESM1_ESM.pdf]

Using quantitative systems pharmacology to evaluate the drug efficacy of COX-2 and 5-LOX inhibitors in therapeutic situations

Authors: Christoph Thiel[a], Ines Smit[b], Vanessa Baier[a], Henrik Cordes[a], Brigida Fabry[a], Lars Mathias Blank[a], and Lars Kuepfer[a]

Affiliations: [a]Institute of Applied Microbiology (iAMB), Aachen Biology and Biotechnology (ABBt), RWTH Aachen University, Worringerweg 1, 52074 Aachen, Germany

[b]European Molecular Biology Laboratory, European Bioinformatics Institute (EMBL-EBI), Wellcome Genome Campus, Hinxton, Cambridge CB10 1SD, UK

Corresponding author: Lars Kuepfer, RWTH Aachen, Worringer Weg 1, 52074 Aachen, Germany, e-mail: lars.kuepfer@rwth-aachen.de, telephone number: +49(0)2418026605, fax number: +49(0)24180622180

SUPPLEMENTARY INFORMATION

PBPK model development

Properties of diclofenac, celecoxib, zileuton, licofelone, rifampicin and their metabolites are identified in literature and are used to establish the human PBPK models (Supplementary Table S2). In some cases, logP and Fu values as well as initial intestinal permeabilities provided by the modeling software[1] are slightly adjusted to best describe the clinical PK data. To represent the ADME behavior several active transport processes, metabolizing reactions, and clearance processes are considered in the respective model structure (Fig. 2, Supplementary Table S3). Relative abundances of relevant transporters and enzymes are estimated by using tissue-specific gene expression data.[2] Since diclofenac and its metabolites are highly bound to plasma proteins, an endothelial barrier between the plasma and the interstitial space is assumed for all organs (brain: 8.53E-05 cm/s (diclofenac), 8.24E-05 cm/s (3'-hydroxy-diclofenac), 8.79E-05 cm/s (4'-hydroxy-diclofenac), 1.44E-05 cm/s (5-hydroxy-diclofenac), 2.4E-7 cm/s (diclofenac-acyl-glucuronide), other organs/tissues: 0.05 cm/s) except the liver. The best-performing distribution models provided in the modeling software are used to determine intracellular-to-plasma partition coefficients (celecoxib: Rodgers and Rowland[3,4]; diclofenac: PK-Sim Standard; licofelone: PK-Sim Standard; rifampicin: Schmitt[5]; zileuton: PK-Sim Standard) as well as permeabilities between the interstitial and the cellular space (celecoxib: PK-Sim Standard[1]; diclofenac: PK-Sim Standard; licofelone: PK-Sim Standard; rifampicin: charge dependent Schmitt[5]; zileuton: PK-Sim Standard).

Developed PBPK models are validated by comparing simulated drug concentrations with clinical PK data from literature[6–21]. Thereby, PBPK model parameters are kept unchanged, besides parameters specifying the clinical study design (e.g. the dose level). Since CYP2C9 genetic polymorphisms have an influence on the pharmacokinetics of celecoxib, different kinetic parameters are used to take into account the differences in drug plasma concentrations for the CYP2C9 genotypes (Supplementary Table S3).

Model quality is assessed by visually inspecting simulated and experimental drug concentrations. In addition, normalized root-mean-square deviation (RMSD)[22] as well as coefficient of determination (R^2) are calculated, and percentage deviations between simulated and experimental Cmax and AUC($0 \rightarrow \infty$) are evaluated (Supplementary Fig. S1).

SUPPLEMENTARY FIGURE LEGENDS

Supplementary Fig. S1 PBPK model assessment.
Observed vs. predicted plots, normalized RMSD value, coefficient of determination (R2), deviations of Cmax and AUC(0•∞) values determined by comparing experimental PK data with simulated drug concentration-time profiles. References and dose levels of the experimental data are shown above each plot. Root-mean-square deviation, RMSD; area under the curve, AUC; maximal concentration, Cmax; Diclofenac, DFN; hydroxy, OH; acyl glucuronide, AGLU; celecoxib, CEL; carboxy, COOH; glucuronide, GLU; zileuton, ZLT; sulfoxide, SO; licofelone, LCF; rifampicin, RIF; desacetyl, DA.

Supplementary Fig. S2 Rifampicin-induced activation dynamics of CYP3A4.
a Intracellular concentrations of unbound rifampicin are simulated in the liver following oral administration of 600 mg q.d. over one week. b Rifampicin-induced induction of CYP3A4 mRNA expression levels and CYP3A4 enzyme activity. Once daily, “q.d”.;

Supplementary Fig. S3 Conceptual overview of the QSP PBPK/PD approach.
PBPK models in-house developed by use of PK-Sim are coupled with existing SBML models from literature by using the MoBi toolbox for MATLAB and the IQM toolbox to perform PBPK/PD simulations.

REFERNCES

1. Willmann, S. *et al.* PK-Sim®: a physiologically based pharmacokinetic 'whole-body' model. *Biosilico* **1,** 121–124 (2003).

2. Meyer, M., Schneckener, S., Ludewig, B., Kuepfer, L. & Lippert, J. Using expression data for quantification of active processes in physiologically based pharmacokinetic modeling. *Drug Metab. Dispos.* **40,** 892–901 (2012).

3. Rodgers, T. & Rowland, M. Physiologically based pharmacokinetic modelling 2: predicting the tissue distribution of acids, very weak bases, neutrals and zwitterions. *J. Pharm. Sci.* **95,** 1238–57 (2006).

4. Rodgers, T., Leahy, D. & Rowland, M. Physiologically based pharmacokinetic modeling 1: predicting the tissue distribution of moderate-to-strong bases. *J. Pharm. Sci.* **94,** 1259–76 (2005).

5. Schmitt, W. General approach for the calculation of tissue to plasma partition coefficients. *Toxicol Vitr.* **22,** 457–467 (2008).

6. Awni, W. M. *et al.* Pharmacokinetics of Zileuton and Its Metabolites in Patients with Renal Impairment. *J. Clin. Pharmacol.* **37,** 395–404 (1997).

7. Crook, P. R., Willis, J. V., Kendall, M. J., Jack, D. B. & Fowler, P. D. The pharmacokinetics of diclofenac sodium in patients with active rheumatoid disease. *Eur. J. Clin. Pharmacol.* **21,** 331–334 (1982).

8. Houin, G. *et al.* Pharmacokinetics of rifampicin and desacetylrifampicin in tuberculous patients after different rates of infusion. *Ther. Drug Monit.* **5,** 67–72 (1983).

9. Braeckman, R. A. *et al.* The Pharmacokinetics of Zileuton in Healthy Young and Elderly Volunteers. *Clin. Pharmacokinet.* **29,** 42–48 (1995).

10. Ratti, B., Parenti, R. R., Toselli, A. & Zerilli, L. F. F. Quantitative assay of rifampicin and its main metabolite 25-desacetylrifampicin in human plasma by reversed-phase high-performance liquid chromatography. *J. Chromatogr. B Biomed. Sci. Appl.* **225,** 526–531 (1981).

11. Paulson, S. K. *et al.* Pharmacokinetics of celecoxib after oral administration in dogs and humans: effect of food and site of absorption. *J. Pharmacol. Exp. Ther.* **297,** 638–645 (2001).

12. Wong, S. L. *et al.* The Pharmacokinetics of Single Oral Doses of Zileuton 200 to 800mg, its Enantiomers, and its Metabolites, in Normal Healthy Volunteers. *Clin. Pharmacokinet.* **29,** 9–21 (1995).

13. Paulson, S. K. *et al.* Metabolism and excretion of [14C]celecoxib in healthy male volunteers. *Drug Metab. Dispos.* **28,** 308–314 (2000).

14. Zhang, Y. *et al.* Diclofenac and Its Acyl Glucuronide: Determination of in Vivo Exposure in Human Subjects and Characterization as Human Drug Transporter Substrates in Vitro. *Drug Metab. Dispos.* **44,** 320–328 (2016).

15. Degen, P. H., Dieterle, W., Schneider, W., Theobald, W. & Sinterhauf, U. Pharmacokinetics of Diclofenac and Five Metabolites After Single Doses in Healthy Volunteers and After Repeated Doses in Patients. *Xenobiotica.* **18,** 1449–1455 (1988).

16. Acocella, G. Clinical pharmacokinetics of rifampicin. *Clin. Pharmacokinet.* **3,** 108–127 (1978).

17. Kirchheiner, J. *et al.* Influence of CYP2C9 genetic polymorphisms on pharmacokinetics of celecoxib and its metabolites. *Pharmacogenetics* **13,** 473–80 (2003).

18. Agrawal, S. *et al.* Comparative bioavailability of rifampicin, isoniazid and pyrazinamide from a four drug fixed dose combination with separate formulations at the same dose levels. *Int. J. Pharm.* **276,** 41–49 (2004).

19. Vergez, J. A., Faour, J., Ricci, M. A. & Befumo, M. E. Osmotic device containing licofelone. *United States Pat. Appl. Publ.* **0129764 A1,** (2005).

20. FDA. Food and Drug Administration. Drugs@FDA http://www.accessdata.fda.gov/drugsatfda_docs/label/2010/050420s073,050627s012lbl.pdf [Accessed 29 September 2015]. (2015).

21. Willis, J. V. V, Kendall, M. J. J., Flinn, R. M. M., Thornbill, D. P. P. & Welling, P. G. G. The pharmacokinetics of diclofenc sodium following intravenous and oral administration. *Eur. J. Clin Pharmacol* **16,** 405–410 (1979).

22. Thiel, C. *et al.* A systematic evaluation of the use of physiologically based pharmacokinetic modeling for cross-species extrapolation. *J. Pharm. Sci.* **104,** 191–206 (2015).
