# Supplementary material for: Using quantitative systems pharmacology to evaluate the drug efficacy of COX-2 and 5-LOX inhibitors in therapeutic situations
Source: NPJ Syst Biol Appl. 2018 Aug 3;4:28. doi: 10.1038/s41540-018-0062-3 (PMC6072773; doi:10.1038/s41540-018-0062-3)
Supplement: Supplementary file 2 — Supplementary Table S1 [file 41540_2018_62_MOESM2_ESM.pdf]

Supplementary Table S1. Potential use of the PBPK/PD QSP approach in drug development.

Potential use of the presented PBPK/PD QSP approach in different phases of the drug development process. The conditions to apply the approach and the potential usage are additionally presented.

Phase	Conditions	Usage
**Lead optimization/ Preclinical**	- Target identification + Network modeling<br>- In vitro binding data (e.g. IC50)<br>- Physicochemistry, PPB, ADME screens	Efficacy
	- Adverse outcome pathway<br>- 2nd target, IC50 of 2nd target<br>- In vitro toxicity data	On-target toxicity<br>Off-target toxicity
**Preclinical**	- Animal in vivo PK data<br>- In vivo PD data	Optimize dosing
**First in human**	- In vitro binding data (e.g. IC50)	Efficacy
**Clinical**	- Human in vivo PK data<br>- Clinical outcome data	Optimize dosing
	- CYP inducer/inhibitor<br>- ADME/PK perpetrator drug	Clinical DDI study
