# Supplementary material for: Using quantitative systems pharmacology to evaluate the drug efficacy of COX-2 and 5-LOX inhibitors in therapeutic situations
Source: NPJ Syst Biol Appl. 2018 Aug 3;4:28. doi: 10.1038/s41540-018-0062-3 (PMC6072773; doi:10.1038/s41540-018-0062-3)
Supplement: Supplementary file 3 — Supplementary Table S2 [file 41540_2018_62_MOESM3_ESM.pdf]

Supplementary Table S2. Properties of the drugs and their metabolites used in the PBPK models.

Molecular weight (MW), octanol/water partition coefficient (logP), fraction unbound (Fu), acid dissociation constant (pKa), water solubility, and intestinal permeability via the transcellular route. Molecular weights, logP, Fu, and pkA values, as well as water solubilities are taken from DrugBank[1], HMDB[2], or were predicted by use of the software ChemAxon[3] or ALOGPS[4]. In some cases, logP ($logP_{initial}$) and Fu values ($Fu_{initial}$) as well as initial intestinal permeabilities provided by the modeling software[5] (Intestinal $permeability_{calc}$) are slightly adjusted ($logP_{model}$, Fu_{model}, Intestinal $permeability_{model}$) to best describe the clinical PK data. Diclofenac, DFN; hydroxy, OH; acyl glucuronide, AGLU; celecoxib, CEL; carboxy, COOH; glucuronide, GLU; zileuton, ZLT; sulfoxide, SO; licofelone, LCF; rifampicin, RIF; desacetyl, DA.

ID	Drug/Metabolite	MW [g/mol]	$logP_{initial}$	$logP_{model}$	$Fu_{initial}$	Fu_{model}	Compound type	pkA	Water Solubility [mg/l]	Intestinal $permeability_{calc}$ [dm/min]	Intestinal $permeability_{model}$ [dm/min]
1	CEL	381.37	3.90	3.70	0.03	0.017	acid	10.70	3.30	3.60E-05	9.00E-05
1	OH-CEL	397.37	3.10	3.30	n/a	0.043	acid	10.70	4.50	1.19E-05	1.19E-05
1	COOH-CEL	411.35	3.24	3.30	n/a	0.039	acid	3.98	3.20	9.95E-06	9.95E-06
2	DFN	296.15	4.51	3.54	<0.003[§]	0.0022	acid	4.15	2.37	8.58E-05	9.37E-06
2	3'-OH-DFN	312.15	3.96	3.68	n/a	0.0018	acid	3.82	10.00	9.40E-05	9.40E-05
2	4'-OH-DFN	312.15	3.96	3.82	n/a	0.0034	acid	3.76	9.60	1.27E-04	1.27E-04
2	5-OH-DFN	312.15	3.96	2.98	n/a	0.0021	acid	3.81	11.00	1.83E-05	1.83E-05
2	DFN-AGLU	472.27	2.31	2.14	n/a	0.0041	acid	3.23	35.70	3.23E-07	3.23E-07
3	LCF	379.88	5.39	4.04	n/a	0.0068*	acid	4.83	1.11	1.00E-03	8.50E-04
3	LCF-AGLU	511.00	4.37	4.37	n/a	0.0841**	[acid, base]	[3.8, 8.9]	1.46	2.96E-05	2.96E-05
3	OH-LCF	395.88	5.42	5.42	n/a	0.0232**	acid	4.64	1.58	1.00E-03	1.00E-03
4	RIF	822.94	2.70	2.92	0.2[$]	0.15	[acid, base]	[1.7, 7.9]	1400	1.01E-07	1.00E-05
4	DA-RIF	780.90	2.57	2.10	n/a	0.05	[acid, base]	[6.9, 7.9]	48.00	1.90E-08	5.00E-05
5	ZLT	236.29	0.90	1.40	0.07	0.095	acid	8.84	500.00	8.46E-07	4.20E-05
5	ZLT-SO	252.29	0.40	0.40	n/a	0.069	acid	8.92	622.00	6.23E-08	6.23E-08
5	OH-ZLT	252.29	1.76	1.76	n/a	0.069	acid	8.73	84.00	1.43E-06	1.43E-06
5	ZLT-GLU	412.414	0.07	-0.50	n/a	0.205	[acid, base]	[4.05, 3.06]	147.00	8.59E-10	8.59E-10

* fitted to experimental data

** predicted by Gertz et al.[6]

§ Davies et al.[7]

$ Acocella et al.[8]

REFERENCES

1. Wishart, D. S. *et al.* DrugBank: a comprehensive resource for in silico drug discovery and exploration. *Nucleic Acids Res* **34,** D668--D672 (2006).
2. Wishart, D. S. *et al.* HMDB: the Human Metabolome Database. *Nucleic Acids Res.* **35,** D521-6 (2007).
3. Swain, M. chemicalize.org chemicalize.org by ChemAxon Ltd. *J. Chem. Inf. Model.* **52,** 613–615 (2012).
4. Tetko, I. V. & Tanchuk, V. Y. Application of Associative Neural Networks for Prediction of Lipophilicity in ALOGPS 2.1 Program. *J. Chem. Inf. Comput. Sci.* **42,** 1136–1145 (2002).
5. Willmann, S. *et al.* PK-Sim®: a physiologically based pharmacokinetic 'whole-body' model. *Biosilico* **1,** 121–124 (2003).
6. Gertz, M., Kilford, P. J., Houston, J. B. & Galetin, A. Drug lipophilicity and microsomal protein concentration as determinants in the prediction of the fraction unbound in microsomal incubations. *Drug Metab. Dispos.* **36,** 535–542 (2008).
7. Davies, N. M. & Anderson, K. E. Clinical pharmacokinetics of diclofenac. Therapeutic insights and pitfalls. *Clin. Pharmacokinet.* **33,** 184–213 (1997).
8. Acocella, G. Clinical pharmacokinetics of rifampicin. *Clin. Pharmacokinet.* **3,** 108–127 (1978).
