# Supplementary material for: Using quantitative systems pharmacology to evaluate the drug efficacy of COX-2 and 5-LOX inhibitors in therapeutic situations
Source: NPJ Syst Biol Appl. 2018 Aug 3;4:28. doi: 10.1038/s41540-018-0062-3 (PMC6072773; doi:10.1038/s41540-018-0062-3)
Supplement: Supplementary file 4 — Supplementary Table S3 [file 41540_2018_62_MOESM4_ESM.pdf]

Supplementary Table S3. ADME processes implemented in the PBPK models.

Metabolizing reactions, active transport processes (influx, efflux), and clearance processes (renal, biliary, hepatic) that were implemented in the PBPK models to represent specific ADME behavior. In case of CEL and ZLT, renal clearance is represented by glomerular filtration (GFR), and GFR and tubular secretion, respectively. Diclofenac, DFN; hydroxy, OH; acyl glucuronide, AGLU; celecoxib, CEL; carboxy, COOH; glucuronide, GLU; zileuton, ZLT; sulfoxide, SO; licofelone, LCF; rifampicin, RIF; desacetyl, DA.

ID	Drug / Metabolite	Metabolite / transporter type / clearance process	Metabolic enzyme / transporter / clearance type	Km [µmol/l]	kcat / specific clearance [1/min]*
1	CEL	OH-CEL	CYP2C9 (zero 2C9*3)[1]	5.1[1]	9.4
1	CEL	OH-CEL	CYP2C9 (one 2C9*3)[1]	5.9[1]	7.1
1	CEL	OH-CEL	CYP2C9 (two 2C9*3)[1]	11.0[1]	4.0
1	OH-CEL	COOH-CEL	ADH2[1]	10.0[1]	54.7
1	COOH-CEL	Hepatic	UGT1A1[2]	100.5*	326.4
1	CEL	Renal[2]	Glomerular filtration[3]	/	8.00**
2	DFN	3’-OH-DFN	CYP2C9[4]	9.0[5]	1.5
2	DFN	4’-OH-DFN	CYP2C9[4]	9.0[5]	67.7
2	DFN	5-OH-DFN	CYP3A4[6]	43.0[5]	6.8
2	DFN	DFN-AGLU	UGT2B7[6]	105.9*	14232.1
2	DFN	Influx	OAT2[7]	48.8[7]	19482.5
2	DFN-AGLU	Influx	OAT2[7]	8.6[7]	3696.3
2	DFN	Renal[2]	Plasma clearance	/	0.008
2	DFN-AGLU	Renal[7]	Plasma clearance	/	315.8
2	3’-OH-DFN	Hepatic[6,2]	Plasma clearance	/	2.8
2	4’-OH-DFN	Hepatic[6,2]	Plasma clearance	/	25.6
2	5-OH-DFN	Hepatic[6,2]	Plasma clearance	/	15.3
3	LCF	LCF-AGLU	UGT2B7[6,8,9]	10.0	340.6
3	LCF	OH-LCF	CYP2J2[6,8,9]	10.0	49.0
3	LCF	Renal[10]	Plasma clearance	/	15.8
3	LCF-AGLU	Hepatic[6]	Plasma clearance	/	4905.0
3	OH-LCF	Hepatic[6]	Plasma clearance	/	4922.4
4	RIF	Influx	OATP-C[11]	1.21[11]	0.08
4	RIF	Efflux	ABCB1[12]	68.5*	21.5
4	RIF	DA-RIF	CES2[13]	53.0*	1.2
4	RIF	Renal[14]	Plasma clearance	/	0.05
4	DA-RIF	Biliary[15]	Plasma clearance	/	0.2
4	DA-RIF	Renal[15]	Plasma clearance	/	3.8
5	ZLT	ZLT-SO	CYP3A4[2]	0.8*	0.4
5	ZLT	OH-ZLT	CYP1A2[2]	0.4*	0.2
5	ZLT	ZLT-GLU	UGT1A9[2]	0.9*	10056.9
5	ZLT-SO	Renal[16]	Tubular secretion[16]	/	6.2
5	OH-ZLT	Renal[16]	Tubular secretion[16]	/	6.2
5	ZLT-GLU	Renal[16]	Tubular secretion[16]	/	2.5
5	ZLT-SO	Renal[16]	Glomerular filtration[16]	/	1.3**
5	OH-ZLT	Renal[16]	Glomerular filtration[16]	/	1.3**
5	ZLT-GLU	Renal[16]	Glomerular filtration[16]	/	1.3**

* fitted to experimental data

** [GFR fraction]

REFERENCES

1. Sandberg, M., Yasar, Ü., Strömberg, P., Höög, J. O. & Eliasson, E. Oxidation of celecoxib by polymorphic cytochrome P450 2C9 and alcohol dehydrogenase. *Br. J. Clin. Pharmacol.* **54,** 423–429 (2002).
2. Wishart, D. S. *et al.* DrugBank: a comprehensive resource for in silico drug discovery and exploration. *Nucleic Acids Res* **34,** D668--D672 (2006).
3. Davies, N. M., McLachlan, A. J., Day, R. O. & Williams, K. M. Clinical Pharmacokinetics and Pharmacodynamics of Celecoxib. *Clin. Pharmacokinet.* **38,** 225–242 (2000).
4. Yasar, Ü. *et al.* The role of CYP2C9 genotype in the metabolism of diclofenac in vivo and in vitro. *Eur. J. Clin. Pharmacol.* **57,** 729–735 (2001).
5. Bort, R. *et al.* Hepatic metabolism of diclofenac: role of human CYP in the minor oxidative pathways. *Biochem. Pharmacol.* **58,** 787–796 (1999).
6. Zhou, S. F., Zhou, Z. W., Yang, L. P. & Cai, J. P. Substrates, inducers, inhibitors and structure-activity relationships of human cytochrome P450 2C9 and implications in drug development. *Curr. Med. Chem.* **16,** 3480–3675 (2009).
7. Zhang, Y. *et al.* Diclofenac and Its Acyl Glucuronide: Determination of in Vivo Exposure in Human Subjects and Characterization as Human Drug Transporter Substrates in Vitro. *Drug Metab. Dispos.* **44,** 320–328 (2016).
8. Gao, J., Fan, H. & Mei, L. Asian Journal of Pharmacodynamics and Pharmacokinetics Pharmacological activities and pharmacokinetics of licofelone. **2010,** 287–299 (2010).
9. Albrecht, W., Unger, A., Nussler, A. K. & Laufer, S. In Vitro Metabolism of 2- [6- (4-Chlorophenyl) -2 , 2-dimethyl-7- phenyl-2 , 3-dihydro-1 H -pyrrolizin-5-yl] Acetic Acid (Licofelone , ML3000), an Inhibitor of Cyclooxygenase-1 and -2 and 5-Lipoxygenase. **36,** 894–903 (2008).
10. Deigner, H. P., Freyberg, C. E. & Laufer, S. Distribution and excretion of [14C]-labelled [2,2-dimethyl-6-(4-chlorophenyl)-7-phenyl-2,3-dihydro-1H-pyrrolizine-5- yl]- [2'-14C]-acetic acid in rats. *Arzneimittelforschung.* **45,** 272–6 (1995).
11. Tirona, R. G. *et al.* Human Organic Anion Transporting Polypeptide-C (SLC21A6) Is a Major Determinant of Rifampin-Mediated Pregnane X Receptor Activation. *Pharmacology* **304,** 223–228 (2003).
12. Williamson, B., Dooley, K. E., Zhang, Y., Back, D. J. & Owen, A. Induction of influx and efflux transporters and cytochrome P450 3A4 in primary human hepatocytes by rifampin, rifabutin, and rifapentine. *Antimicrob. Agents Chemother.* **57,** 6366–6369 (2013).
13. Song, S. H. *et al.* Relationship between CES2 genetic variations and rifampicin metabolism. *J. Antimicrob. Chemother.* **68,** 1281–1284 (2013).
14. FDA. Food and Drug Administration. Drugs@FDA http://www.accessdata.fda.gov/drugsatfda_docs/label/2010/050420s073,050627s012lbl.pdf [Accessed 29 September 2015]. (2015).
15. Acocella, G. Clinical pharmacokinetics of rifampicin. *Clin. Pharmacokinet.* **3,** 108–127 (1978).
16. Wong, S. L. *et al.* The Pharmacokinetics of Single Oral Doses of Zileuton 200 to 800mg, its Enantiomers, and its Metabolites, in Normal Healthy Volunteers. *Clin. Pharmacokinet.* **29,** 9–21 (1995).
