# Supplementary material for: Using quantitative systems pharmacology to evaluate the drug efficacy of COX-2 and 5-LOX inhibitors in therapeutic situations
Source: NPJ Syst Biol Appl. 2018 Aug 3;4:28. doi: 10.1038/s41540-018-0062-3 (PMC6072773; doi:10.1038/s41540-018-0062-3)
Supplement: Supplementary file 5 — Supplementary Table S4 [file 41540_2018_62_MOESM5_ESM.pdf]

Supplementary Table S4. Model evaluation.

Evaluation of different models used to describe predicted prostaglandin decrease and mean pain relief observed in patients. SSE, RMSE, R^2_{adj} are calculated by use of the curve fitting toolbox of MATLAB. AICc and BIC are calculated according to Spiess et al.[1] The three-parameter hill model can be found in Goutelle et al.[2] Diclofenac, DFN; celecoxib, CEL; bias-corrected Akaike Information Criterion, AICc; Bayesian Information Criterion, BIC; Sum of squares due to error, SSE; Root mean squared error, RMSE; Degree-of-freedom adjusted coefficient of determination, R^2_{adj}; number of parameters, #p.

**Drug (time)**	**Model (#p)**	**AICc**	**BIC**	**SSE**	**RMSE**	**R^2_{adj}**
DFN (6h)	Linear (2)	15.88	18.47	2.74	0.30	0.86
DFN (6h)	Hill (3)	-6.27	-2.61	1.30	0.21	0.93
DFN (6h)	Quadratic (3)	3.67	7.34	1.76	0.24	0.91
CEL (6h)	Linear (2)	9.85	9.15	0.96	0.33	0.85
CEL (6h)	Hill (3)	-13.14	-15.37	0.08	0.10	0.98
CEL (24h)	Linear (2)	33.1	34.23	4.95	0.54	0.32
CEL (24h)	Hill (3)	1.25	2.48	0.80	0.22	0.88

REFERENCES

1. Spiess, A. N. & Neumeyer, N. An evaluation of R2 as an inadequate measure for nonlinear models in pharmacological and biochemical research: A Monte Carlo approach. *BMC Pharmacol.* **10,** 1–11 (2010).
2. Goutelle, S. *et al.* The Hill equation: A review of its capabilities in pharmacological modelling. *Fundam. Clin. Pharmacol.* **22,** 633–648 (2008).
