# Supplementary figures and images for: Using quantitative systems pharmacology to evaluate the drug efficacy of COX-2 and 5-LOX inhibitors in therapeutic situations
Source: NPJ Syst Biol Appl. 2018 Aug 3;4:28. doi: 10.1038/s41540-018-0062-3 (PMC6072773; doi:10.1038/s41540-018-0062-3)

predicted concentration [ $\mu\text{mol/l}$ ]

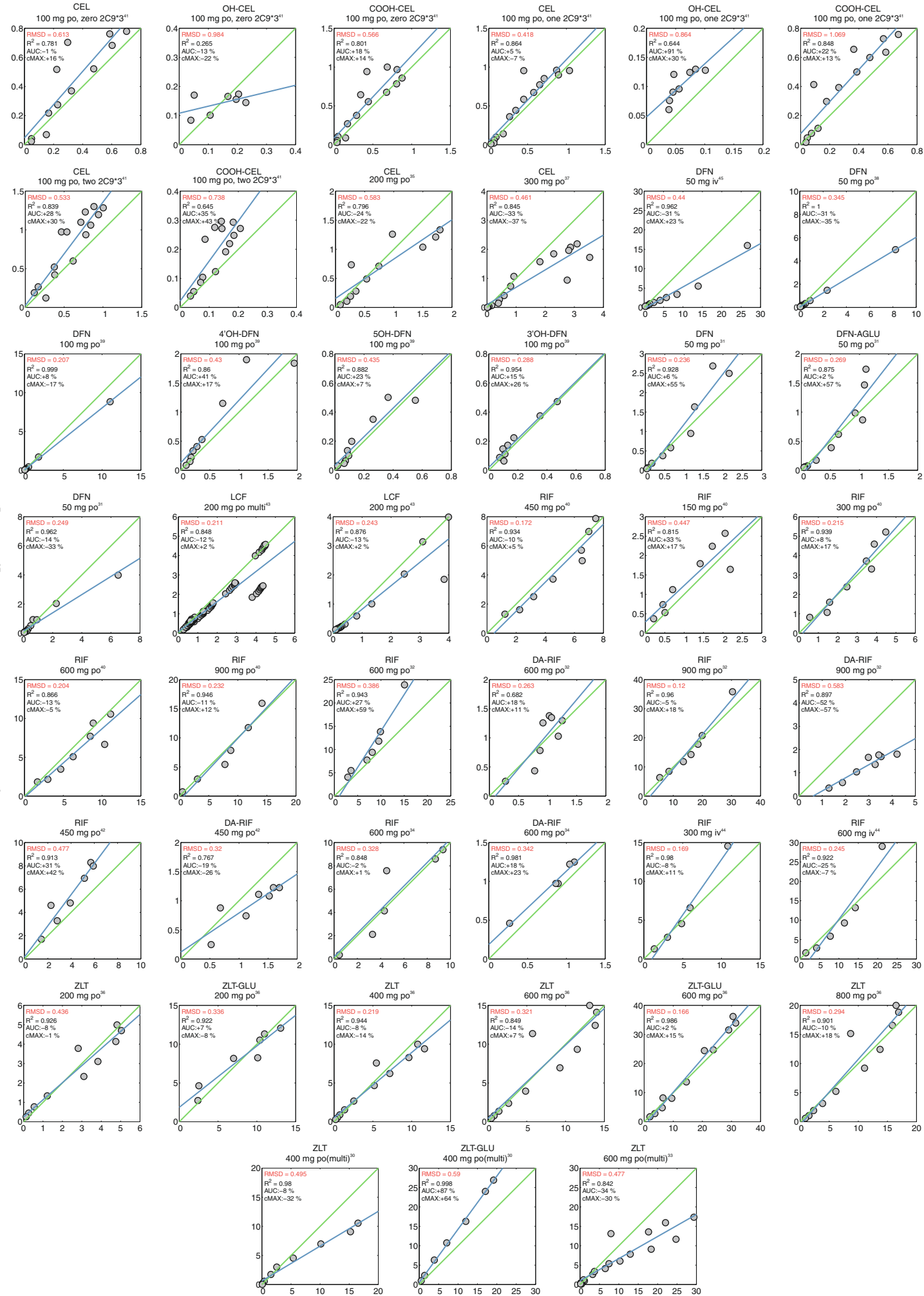

observed concentration [ $\mu\text{mol/l}$ ]

Supplement: Supplementary file 6 — Supplementary Fig. S1 [file 41540_2018_62_MOESM6_ESM.pdf]

**a**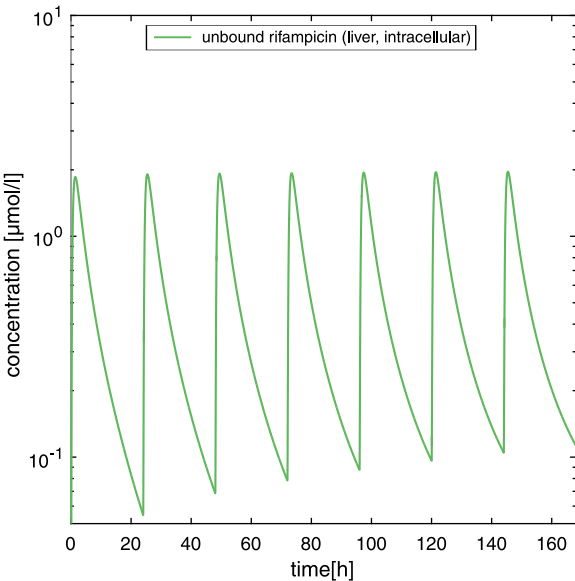**b**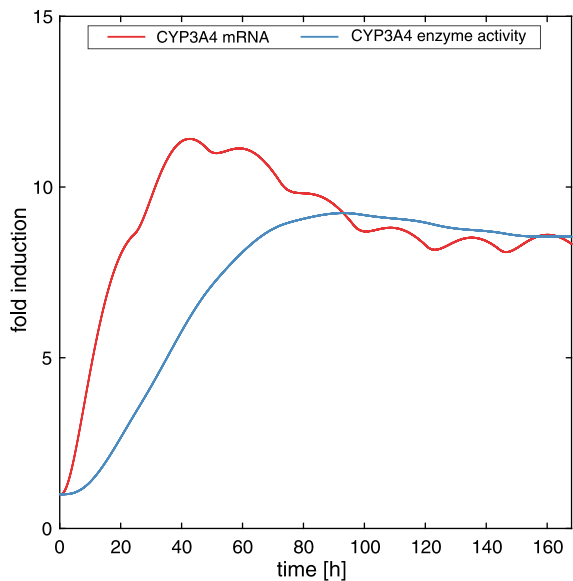

Supplement: Supplementary file 7 — Supplementary Fig. S2 [file 41540_2018_62_MOESM7_ESM.pdf]
