# Supplementary material for: Using quantitative systems pharmacology to evaluate the drug efficacy of COX-2 and 5-LOX inhibitors in therapeutic situations
Source: NPJ Syst Biol Appl. 2018 Aug 3;4:28. doi: 10.1038/s41540-018-0062-3 (PMC6072773; doi:10.1038/s41540-018-0062-3)
Supplement: Supplementary file 8 — Supplementary Fig. S3 [file 41540_2018_62_MOESM8_ESM.pdf]

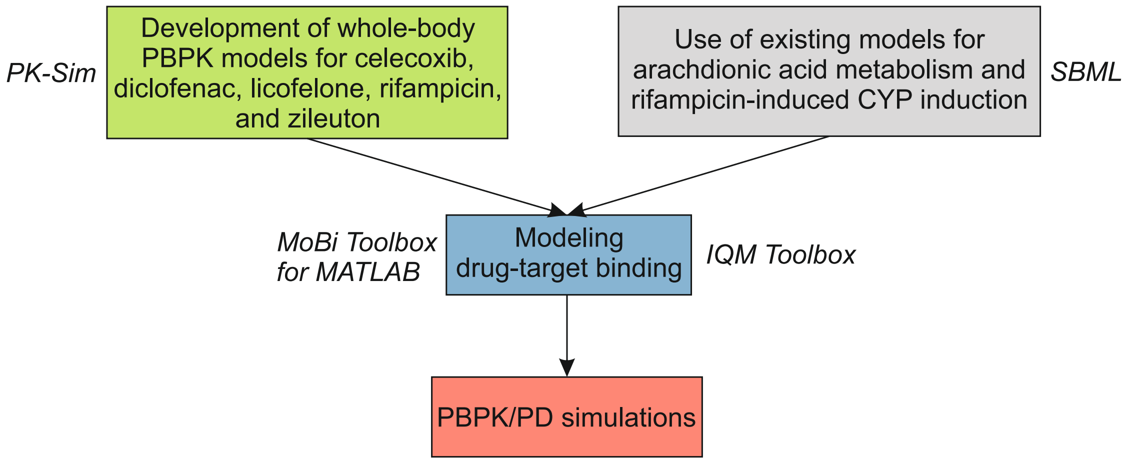
PK-Sim
Development of whole-body PBPK models for celecoxib, diclofenac, licofelone, rifampicin, and zileuton
Use of existing models for arachdionic acid metabolism and rifampicin-induced CYP induction
SBML
MoBi Toolbox for MATLAB
Modeling drug-target binding
IQM Toolbox
PBPK/PD simulations
